# Preliminary In Vitro Study of Fluoride Release from Selected Ormocer Materials

**DOI:** 10.3390/ma14092244

**Published:** 2021-04-27

**Authors:** Piotr Kosior, Maciej Dobrzynski, Aneta Zakrzewska, Lukasz Grosman, Mariusz Korczynski, Tomasz Blicharski, Martina Gutbier, Adam Watras, Rafal J. Wiglusz

**Affiliations:** 1 Department of Conservative Dentistry with Endodontics, Wroclaw Medical University, Krakowska 26, 50-425 Wroclaw, Poland; piotr.kosior@umed.wroc.pl; 2 Department of Pediatric Dentistry and Preclinical Dentistry, Wroclaw Medical University, Krakowska 26, 50-425 Wroclaw, Poland; martina.gutbier@umed.wroc.pl; 3 Department of Periodontology, Wroclaw Medical University, Krakowska 26, 50-425 Wroclaw, Poland; aneta.zakrzewska@umed.wroc.pl; 4 Institute of Low Temperature and Structure Research, Polish Academy of Sciences, Okolna 2, 50-422 Wroclaw, Poland; l.grosman@intibs.pl; 5 Department of Animal Nutrition and Feed Management, Wroclaw University of Environmental and Life Sciences, Chelmonskiego 38C, 51-630 Wroclaw, Poland; mariusz.korczynski@upwr.edu.pl; 6 Department of Rehabilitation and Orthopedics, Medical University of Lublin, Jaczewskiego 8, 20-954 Lublin, Poland; blicharski@vp.pl

**Keywords:** fluoride ion release, ormocer materials, fluoride recharge

## Abstract

The purpose of the in vitro study presented in this paper was to determine the long-term release of fluoride ions from selected ormocer materials (Admira (A), Admira Flow (AF), Admira Seal (AS)). The release of fluoride ions from these materials into a saline solution (0.9% NaCl) and deionized water was tested for 14 weeks. In a long-term study the measurements were taken after 1 and 3 h, then 1, 2, and 3 days and then at weekly intervals for 14 weeks. In a short-term study the measurements were made after 3, 24, 48, 72, 69, 168 h, i.e., within 7 days. All materials used in the test showed a constant level of fluoride release. The highest level of cumulative release of fluoride ions into deionized water was found in the AS material (23.95 ± 4.30 μg/mm^2^), slightly lower in the A material (23.26 ± 4.16 μg/mm^2^) and the lowest in the AF material (16.79 ± 2.26 μg/mm^2^). The highest level of cumulative release into saline solution was found in AF (8.08 ± 1.30 μg/mm^2^), slightly lower in AS (7.36 ± 0.30 μg/mm^2^) and the lowest in A (6.73 ± 1.10 μg /mm^2^). After 1 h of immersion of the samples in the saline solution, the highest level of fluoride was released by AF (0.57 ± 0.06 μg/mm^2^) followed by A (0.20 ± 0.03 μg/mm^2^) and AS (0.19 ± 0.02 µg/mm^2^). Moreover, in the 14-week study, the total amount of fluoride release into the saline, which imitates the environment of the oral cavity, was observed as the highest in the AF sample.

## 1. Introduction

The high reactivity of fluoride is related to its multidirectional influence on cariogenic processes [1,2,3,4]. Fluoride ions show antibacterial effects and help maintain the mineral balance of dental hard tissues. The bacteriostatic effect is possible due to the fact that fluoride in the form of hydrofluoric acid passes through the cell membrane of bacteria, where it inhibits the enolase, a glycolytic enzyme important for bacterial metabolism. The released fluoride ions are also involved in blocking the glucose transport, affecting the proper functioning of bacteria and thus inhibiting the growth of dental plaque [5].

Fluoride ions counteract the carious processes of dental plaque [2]. Their proven cariostatic effect is associated with the possibility of modifying enamel hydroxyapatite. The mechanism of reaction is based on the replacement of the hydroxide ions of enamel hydroxyapatite with fluoride ions, resulting in the formation of fluorapatites [6]. Fluorapatite is characterized by superior crystalline properties due to strong ionic bonds between fluoride and NH groups of the organic enamel matrix; it is also less soluble in an acidic medium.

As a result of frequent exposure to low fluoride concentrations, enamel remineralization is enhanced, whereas demineralization processes are inhibited [7]. This proven action of fluoride is used by manufacturers of restorative materials in the prevention of tooth decay in molars and premolars [8,9,10,11,12,13,14,15,16]. The ormocer group is an example of such materials. The name ormocer is an acronym for organically modified ceramics. These materials were developed as special technical coatings, such as antistatic or non-stick coatings, at the Fraunhofer Institute in Würzburg, Germany. Currently, they are used clinically as dental restorative materials due to their reduced shrinkage as well as having good aesthetics and abrasive properties [17].

Compared to standard composite materials for the restoration of teeth, ormocer polymer systems are made of alkoxy silicates, i.e., RnSi(OR′)(4 − n) particles. The inorganic center contains oxygen and silicon atoms, and the organic parts are multifunctional urethane and thioether methacrylate groups, containing double carbon–carbon bonds, through which the ormocer is cured by additive polymerization. These organic parts are precursors of sol-gel. Silane alkoxy silane groups enable the formation of an inorganic Si-O-Si network by hydrolysis and polycondensation reactions [17].

An example of commercial materials based on ormocer systems are the restorative materials produced by Voco: Admira (A), Admira Flow (AF) and Admira Seal (AS). All tested materials contain sodium fluoride in their composition. The purpose of the tests presented in this publication is to determine the level of release of the fluoride that they contain.

## 2. Materials and Methods

Materials used in the research:

- Admira (A), (Voco, Cuxhaven, Germany) has a 78% content of inorganic fillers (by weight) which are a mixture of ceramic glass with an average particle size of 0.7 μm and of pyrogenic silica with the size of about 0.04 μm. The particles are prepared chemically, or silanized, to obtain a good connection between the matrix and the filler. The matrix is an inorganic osmolar copolymer and dimethacrylate mix.
- Admira Flow (AF), (Voco, Cuxhaven, Germany) is a light-curing, liquid osmic material for low-viscosity restorations.
- Admira Seal (AS), (Voco, Cuxhaven, Germany) is a material consisting mainly of borosilicate glass (16%).

**Figure 1:**
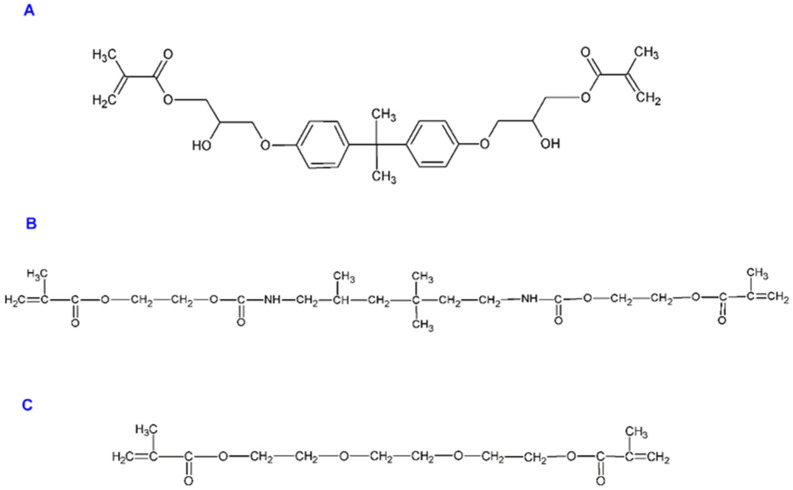
Structures of Bis-GMA (**A**), UDMA (**B**) and TEGDMA (**C**) monomers.

The exact composition of each material is listed in Table 1. The chemical structures of Bis-GMA (A), UDMA (B) and TEGDMA (C) monomers are presented in Figure 1.

Samples of the materials were prepared in the form of a pellet, 5 mm in diameter and 2 mm thick and were made in the shape of cylinders using a polyethylene matrix. The material was polymerized according to the manufacturer’s recommendations. After curing, the samples were polished and conditioned, corresponding to the regular protocol in a clinical setting. Their contact area was calculated. Subsequently they were immersed in the studied solutions and left without stirring in closed containers at 37 °C for a suitable period to determine the fluoride release from the materials. Five samples of each material were prepared for each environment (total *n* = 30). Each sample was examined three times, and an average value was calculated based on the three results.

Release of fluoride ions from these materials into a saline solution (0.9% NaCl) and deionized water was tested for 14 weeks (i.e., 98 days or 2352 h). Deionized water was chosen as a neutral solution completely devoid of ionic strength and the saline solution was to imitate the natural environment of the oral cavity.

The measurement of fluoride ions was performed using ORION model 9609 ion-selective electrodes in combination with a pH/ion meter CPI-551 Elmetron microcomputer. The system was calibrated before each subsequent measurement. The measurement was repeated three times and the calculated mean value was established. In the long-term study the measurements were taken after 1 and 3 h, then 1, 2, and 3 days and then at weekly intervals for 14 weeks. In the short-term study, however, measurements were taken after 3, 24, 48, 72, 69, 168 h, i.e., within 7 days.

After the measurement period, 5 mL of the eluate was taken for evaluation. The sample was dried and transferred to a new solution. The concentrations of fluoride released from the fluoride materials were expressed in μmol/L and in relation to the surface area of the material. The cumulative level of fluoride ion release (i.e., total release over the whole given observation period) and the increments of fluoride release over the given measurement periods and with respect to the unit of time-1 h were determined.

In order to evaluate the physicochemical properties of the materials, the samples were prepared in the form of a pellet, 5 mm in diameter and 2 mm thick. The ormocer resins were cured with the Kulzer Translux EC lamp for 40 s; X-ray Diffraction (XRD) measurements were made on the X’Pert PRO X-ray diffractometer (Cu Kα1, 1.54060 Å) by PANalytical; FT-IR spectra (Fourier Transform Infrared) measurements were performed on a Thermo Scientific Nicolet iS50 FT-IR spectrometer equipped with an ATR module (iS50 ATR). The source of infrared radiation was a HeNe laser.

Statistical analyses were performed by Statisticav.13.3 software (Tibco Software Inc., Palo Alto, CA, USA). Descriptive data were presented as mean, standard deviation (±SD) and 95% confidence interval (±95% CI). Distribution of the data was performed by the Shapiro–Wilk normality test. ANOVA for dependent samples and ANOVA for independent variables were used for the calculation of differences between study groups or subgroups. A post hoc Tukey test was used for inter group comparisons. Any *p*-values of <0.05 were assumed to be statistically significant.

## 3. Results

**Figure 2:**
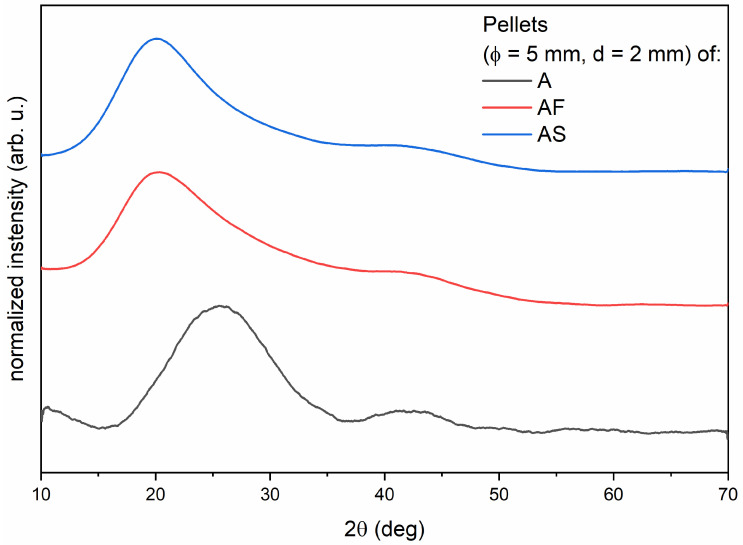
XRD diagrams of ormocer materials. The study was performed for one randomly selected sample of each material: A—Admira; AF—Admira Flow; AS—Admira Seal.

Figure 2 shows the XRD pattern of the Admira pellets light-cured for 40 s. All samples are amorphous, which is demonstrated by a broad diffraction peak centered at 2θ = 26° for material A and 2θ = 20° for materials AF and AS. The difference in position of these peaks is caused by the variable composition of the materials. 

**Figure 3:**
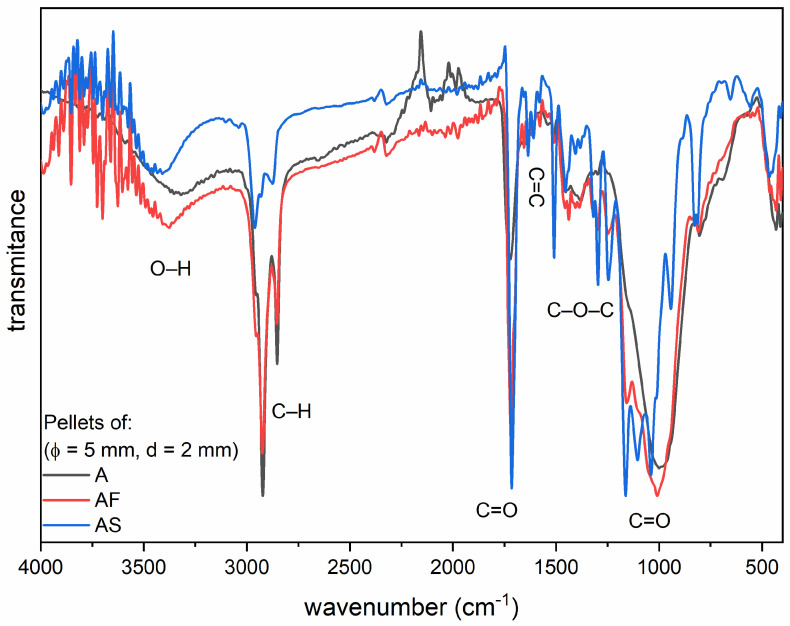
FTIR spectra of ormocer materials light-cured for 40 s. The study was performed for one randomly selected sample of each material: A—Admira; AF—Admira Flow; AS—Admira Seal.

Figure 3 presents the FTIR spectrum which shows the characteristic bands for the Admira resin. The bands with a wavenumber of 1000, 1159 and 1716 cm^−1^ come from the vibrations of the C=O double bond, the band at 1296 cm^−1^ is related to the vibration of the C–O–C molecule, the band located at 1653 cm^−1^ corresponds to the C=C double bond characteristic for dental resins, while intense bands at 2856 and 2920 cm^−1^ are related to the vibrations of the C–H bond. In addition, a weak band is visible around 3500 cm^−1^ associated with the vibration of the O–H group.

Release of fluoride ionsfrom A, AF and AS materials into the saline solution was showed in Table 2.

The highest level of cumulative release was found for material AF (8.08 ± 1.30 μg/mm^2^), slightly lower for material AS (7.36 ± 0.30 μg/mm^2^) and the lowest for material A (6.73 ± 1.10 µg/mm^2^). After 1 h of immersion of the samples in the saline solution, the highest amount of fluoride was released by AF (0.57 ± 0.06 μg/mm^2^) followed by A (0.20 ± 0.03 µg/mm^2^) and AS (0.19 ± 0.02 µg/mm^2^). The highest fluoride release by the A material was observed on the 77th day (1848 h) of incubation, by the AF material—on the 49th day (1176 h) and the 56th day (1344 h), and by the AS material—on the 70th day (1680 h) of incubation. There were statistically significant differences between the release of fluoride ions and the time period of each ormocer material (*p* < 0.0001 for all).

**Figure 4:**
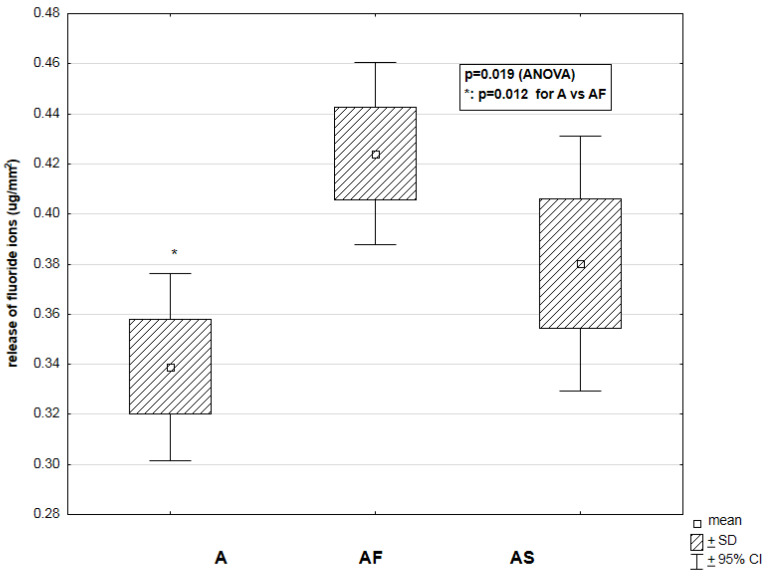
Relationship between the type of ormocer material (A—Admira (*n* = 5); AF—Admira Flow (*n* = 5); AS—Admira Seal (*n* = 5)) and the release of fluoride ions (μg/mm^2^) into the saline solution in a period of 2352 h.

Significant differences were found among the mean values of fluoride release from A, AF and AS materials (*p* = 0.019) (Figure 4). Fluoride release was notably higher in the AF material (0.42 ± 0.18 µg/mm^2^) than in the A group (0.34 ± 0.18 µg/mm^2^) (*p* = 0.012).

Table 3 shows the release of fluoride ions from Admira’s ormocer materials (A, AF, AS) into deionized water. The highest level of cumulative release was found for material AS (23.95 ± 4.30 μg/mm^2^), slightly lower for material A (23.26 ± 4.16 μg/mm^2^) and the lowest for material AF (16.79 ± 2.26 μg/mm^2^). The release of fluoride ions was significantly different in time periods for each of the studied materials (*p* < 0.001).

**Figure 5:**
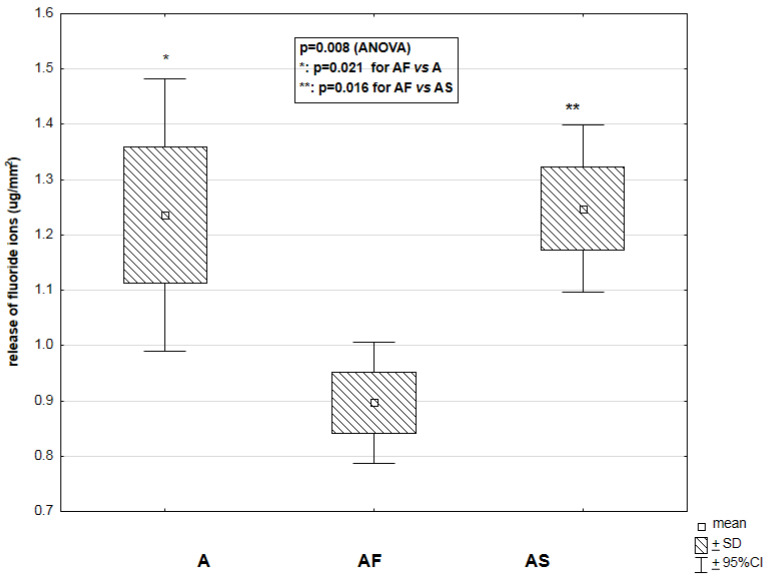
Relationship between the type of studied material (A—Admira (*n* = 5); AF—Admira Flow (*n* = 5); AS—Admira Seal (*n* = 5)) and the release of fluoride ions (μg/mm^2^) into deionized water in a period of 2352 h.

As shown in Figure 5, the ormoceric materials A, AF, and AS demonstrated significantly different means of fluoride release into deionized water (*p* = 0.008). The lowest level of fluoride release was observed in AF samples (0.88 ± 0.52 μg/mm^2^) compared to A (1.22 ± 1.15 μg/mm^2^) and AS (1.26 ± 0.73 μg/mm^2^) groups (*p* < 0.05 for both).

In the oral cavity, the true values of fluoride release will undoubtedly be different due to the variability of the pH of the environment and the presence of other ions.

## 4. Discussion

The materials used in restorative dentistry interact with various elements of the environment within the oral cavity. The fluoride release is higher in deionized water than in artificial or human saliva and saline solution [18,19]. This observation might be explained by the fact that the diffusion gradient between the restorative dental materials and ion-enriched human saliva or saline solution is lower as compared to the gradient between the materials and deionized water [20]. The higher viscosity of artificial or human saliva decreases ions diffusion into and out of materials [21]. In the natural environment of the oral cavity, the presence of other ions contained in the patient’s saliva, can significantly modify the release of fluoride ions from the dental materials surface. In this study the in vitro model was used for several reasons and has its limitation. Firstly, ormocer materials from the Admira group, when exposed to the patient’s oral cavity can release fluoride ions but can also absorb a small amount of fluoride from the surrounding environment. Secondly, this in vitro study eliminates the influence of other factors that may affect the release of fluoride ions, such as saliva pH, the presence of proteins or other ions such as calcium and phosphate ions. Additionally, the cylindrical shape of the tested samples immersed in contact fluids imply that more of the surface of the material is in contact with the ambient fluids and thus more surfaces are available and ready to release fluoride ions. Fluoride release is dependent on the exposed surface area [22]. Whereas in vivo, a material placed in a tooth cavity is generally not exposed to every surface of the oral environment. The choice of contact fluids in which the samples were immersed in, was dictated by the fact that deionized water eliminates the interaction of fluoride ions with other ions and saline is a similar electrolyte compared to the patient’s saliva. Fluid adsorption, mainly taking place in the matrix of the material, is possibly the most interesting aspect of fluoride release from the ormocers. It allows for the penetration of the stable structure of the material by the liquid molecules and for diffusion-controlled fluoride release. The type of resin matrix is a crucial element of the water absorption process of restorative materials. The properties of the matrix determine the diffusion velocity as well as capacity and volume of water absorption in that part of the material [23,24]. The presence of hydrophilic resin monomers (see Figure 1) such as bisphenol A glycerolate dimethacrylate (Bis–GMA), triethylene glycol dimethacrylate (TEGDMA) and urethane dimethacrylate (UDMA), which are found in A, AF [25] and AS, significantly increase water absorption in those materials and contribute to a higher release of fluoride ions. 

Monomers have varying hydrophilicity and may be classified in the following order: TEGDMA > Bis-GMA > UDMA [23]. The process of water absorption in ormocers containing UDMA starts with the formation of hydrogen bonds between UDMA polymers and bound water. Next, the intramolecular hydrogen bonds are affected by the free water molecules, which results in increased plasticity and sliding properties of the adjacent polymer chains [26]. The ion release rate depends on the segmental mobility of the polymer chains and velocity of water sorption [27]. Apart from the properties of the matrix, the amount of filler in the material significantly affects the process of water absorption. According to the available literature, the filler load is negatively correlated with water sorption [28], which is consistent with the findings of this study. Table 2 shows the cumulative release of fluoride ions from ormocer materials from Admira group of different consistency and clinical application to the saline solution. After 14 weeks, the highest emission was found for AF (8.08 ± 1.30 μg/mm^2^), lower for AS (7.36 ± 0.30 μg/mm^2^) and the lowest for A (6.73 ± 1.10 μg/mm^2^). The reduction in the filler concentration in flowable material has a direct influence on the modulus of elasticity. Therefore, due to its low modulus of elasticity and high release of fluoride, AF can be used as a liner to act as a stress relief layer [29], and in the case of minimally invasive cavities, fissure sealing and resin composite restoration repair.

Ormocers are described as three dimensionally cross-linked copolymers [30] with a polymerization process that does not leave any residual monomer behind, making the ormocers more biocompatible to surrounding tissue [31]. The obtained results certainly confirm the cariostatic capabilities of ormoceric materials from the Admira group and the concentration levels of fluoride ions released from the surface of these materials can potentially aid in the remineralization of the surrounding tooth tissues. 

## 5. Conclusions

According to the guidelines of scientific societies on caries prevention, long-term release of fluoride in low concentrations is more beneficial than in high doses over a short period of time [32]. This paper presents the results of a 14-week study on the release of fluoride ions from different Admira ormocer materials, that differ in consistency and clinical application between each other. The obtained data and observations showed that the tested materials present a relatively constant level of release of fluoride in this time frame. It is worth noting that in the case of all tested materials the highest level of released fluoride is observed already after 1 h of the study. In addition, the highest total amount of fluoride ions released was observed in the case of AF material, which is likely to reveal the highest cariostatic efficacy in comparison with other tested opaque materials.

## Figures and Tables

**Table 1:** Composition of dental materials.

Material	Manufacturer	Composition
Admira (A)	Voco, Germany	ormocers (10–25%), Bis-GMA (5–10%), urethane di-methacrylate (5–10%), sodium fluoride
Admira Flow (AF)	Voco, Germany	ormocers (10–25%), 1,6-hexanediyl bismoacrylate (10–25%), Bis-GMA (5–10%), urethane di-methacrylate (5–10%), triethylene glycol di-methacrylate (≤2.5%), sodium fluoride
Admira Seal (AS)	Voco, Germany	borosilicate glass (16%), di-methacrylates, silicate fillers, ormocers and additives (with sodium fluoride)

**Table 2:** Release of fluoride ions (µg/mm^2^) from ormocer materials (A—Admira; AF—Admira Flow; AS—Admira Seal) into a saline solution (0.9% NaCl) in a period of 2352 h. Five samples were prepared for each material.

Time (Hours)	A (*n* = 5) (μg/mm^2^) Mean ± SD	AF (*n* = 5) (μg/mm^2^) Mean ± SD	AS (*n* = 5) (μg/mm^2^) Mean ± SD
**1**	0.20 ± 0.03	0.57 ± 0.06	0.19 ± 0.02
**3**	0.14 ± 0.05	0.34 ± 0.02	0.29 ± 0.02
**24**	0.23 ± 0.04	0.20 ± 0.02	0.13 ± 0.02
**48**	0.23 ± 0.03	0.13 ± 0.02	0.10 ± 0.03
**72**	0.11 ± 0.03	0.20 ± 0.03	0.12 ± 0.03
**168**	0.18 ± 0.03	0.23 ± 0.02	0.10 ± 0.03
**336**	0.37 ± 0.03	0.23 ± 0.04	0.28 ± 0.02
**504**	0.09 ± 0.02	0.15 ± 0.02	0.09 ± 0.02
**672**	0.31 ± 0.03	0.48 ± 0.01	1.13 ± 0.08
**840**	0.36 ± 0.03	0.46 ± 0.02	0.36 ± 0.03
**1008**	0.11 ± 0.06	0.43 ± 0.02	0.35 ± 0.02
**1176**	0.43 ± 0.02	0.65 ± 0.05	0.46 ± 0.02
**1344**	0.43 ± 0.02	0.66 ± 0.04	0.50 ± 0.02
**1512**	0.34 ± 0.02	0.50 ± 0.03	0.40 ± 0.02
**1680**	0.63 ± 0.02	0.61 ± 0.02	0.62 ± 0.03
**1848**	0.65 ± 0.02	0.64 ± 0.02	0.59 ± 0.02
**2016**	0.51 ± 0.03	0.55 ± 0.03	0.46 ± 0.03
**2184**	0.52 ± 0.02	0.50 ± 0.02	0.49 ± 0.02
**2352**	0.59 ± 0.02	0.53 ± 0.02	0.54 ± 0.02
**Mean ± SD**	0.34 ± 0.18	0.42 ± 0.18	0.38 ± 0.25
**Cumulative release of F-ions**	6.73 ± 1.10	8.08 ± 1.30	7.36 ± 0.30
***p*-value** **(ANOVA for dependent samples)**	<0.0001 *	<0.0001 *	<0.0001 *

* statistically significant.

**Table 3:** Release of fluoride ions (μg/mm^2^) from ormocer materials (A—Admira; AF—Admira Flow; AS—Admira Seal) into deionized water in a period of 2352 h. Five samples were prepared for each material.

Time (Hours)	A (*n* = 5) (μg/mm^2^) Mean ± SD	AF (*n* = 5) (μg/mm^2^) Mean ± SD	AS (*n* = 5) (μg/mm^2^) Mean ± SD
**1**	0.65 ± 0.02	0.43 ± 0.03	0.48 ± 0.08
**3**	0.44 ± 0.02	0.26 ± 0.03	0.37 ± 0.13
**24**	0.49 ± 0.03	0.35 ± 0.05	0.49 ± 0.07
**48**	0.43 ± 0.04	0.32 ± 0.01	0.44 ± 0.13
**72**	0.46 ± 0.06	0.38 ± 0.05	0.37 ± 0.32
**168**	0.98 ± 0.03	0.65 ± 0.03	1.00 ± 0.18
**336**	0.44 ± 0.01	0.31 ± 0.04	0.29 ± 0.02
**504**	0.94 ± 0.14	0.75 ± 0.06	0.93 ± 0.11
**672**	0.48 ± 0.08	0.82 ± 0.03	1.43 ± 0.11
**840**	0.42 ± 0.02	0.36 ± 0.08	0.68 ± 0.15
**1008**	1.90 ± 0.15	1.59 ± 0.03	1.51 ± 0.1
**1176**	1.25 ± 0.04	1.3 ± 0.28	2.38 ± 1.04
**1344**	1.27 ± 0.09	1.52 ± 0.45	1.92 ± 0.23
**1512**	1.36 ± 0.14	1.66 ± 0.5	1.97 ± 0.2
**1680**	1.48 ± 0.09	1.52 ± 0.13	1.89 ± 0.17
**1848**	1.42 ± 0.07	1.32 ± 0.24	1.87 ± 0.39
**2016**	1.18 ± 0.07	1.03 ± 0.06	1.67 ± 0.52
**2184**	2.26 ± 0.19	1.53 ± 0.11	2.34 ± 0.32
**2352**	5.41 ± 2.87	0.69 ± 0.05	1.92 ± 0.03
**Mean ± SD**	1.22 ± 1.15	0.88 ± 0.52	1.26 ± 0.73
**Cumulative release of F-ions**	23.26 ± 4.16	16.79 ± 2.26	23.95 ± 4.30
***p*-value** **(ANOVA for dependent samples)**	<0.0001 *	<0.0001 *	<0.0001 *

* statistically significant.

## Data Availability

Not applicable.
